# Deep learning–based auto-segmentation and RECIST evaluation after concurrent chemoradiotherapy in locally advanced hepatocellular carcinoma patients

**DOI:** 10.3389/fonc.2026.1775269

**Published:** 2026-03-30

**Authors:** Jinyoung Moon, Minseo Choi, Yejin Kim, Hyungjin Rhee, Sang Joon Park, Jin Sung Kim, Ik Jae Lee

**Affiliations:** 1 Department of Radiation Oncology, Yonsei University College of Medicine, Seoul, Republic of Korea; 2 Department of Medical Physics, Memorial Sloan Kettering Cancer Center, New York, NY, United States; 3 Department of Radiation Oncology, University of Washington and Fred Hutchinson Cancer Center, Seattle, WA, United States; 4 Department of Radiology, Yonsei University College of Medicine, Seoul, Republic of Korea; 5 Department of Radiation Oncology, Heavy Ion Therapy Research Institute, Yonsei Cancer, Seoul, Republic of Korea; 6 Department of Radiation Oncology, Heavy Ion Therapy Research Institute, Yonsei Cancer Center, Yonsei University Health System, Yonsei University College of Medicine, Seoul, Republic of Korea

**Keywords:** auto-segmentation, CCRT, hepatocellular carcinoma, RECIST, treatment follow up

## Abstract

**Background and purpose:**

Hepatocellular carcinoma (HCC) is the third leading cause of cancer-related mortality, and intrahepatic progression after following treatment is common. Accurate tumor evaluation is essential for treatment decisions but remains challenging due to tumor heterogeneity, the background of cirrhotic liver, and treatment-related artifacts. This study investigated the feasibility of a deep learning–based auto-segmentation approach for response evaluation in locally advanced HCC treated with concurrent chemoradiotherapy (CCRT).

**Methods:**

We retrospectively analyzed 83 treatment-naïve patients with locally advanced HCC who underwent definitive CCRT between 2016 and 2021. Tumor contours were manually delineated on pre-treatment (CTpre) and first post-treatment CT (CTpost). A fully convolutional DenseNet (FCD) and an intentional deep overfit learning (IDOL) framework were trained and validated. Performance was assessed using the Dice similarity coefficient (DSC), and RECIST-based diameters were compared between manual and predicted contours.

**Results:**

In the full cohort, the FCD model achieved mean DSCs of 0.53 for CTpre and 0.33 for CTpost, while the IDOL model improved CTpost DSCs to 0.49. In the RECIST cohort (*n* = 63), mean DSCs were 0.61 for CTpre and 0.53 for CTpost using FCD, versus 0.63 for IDOL. For the RECIST cohort (*n* = 14 validation cases), predicted diameters differed by a mean of 9.2 mm from manual values (*p* = 0.032), showing a tendency toward overestimation in peritumoral inflammatory areas. However, RECIST-based response showed high concordance in 13 of 14 cases.

**Conclusions:**

The patient-specific IDOL framework improved auto-segmentation accuracy compared with conventional models and provided reliable data for RECIST-based response assessment. Despite limitations and lack of external validation, this study demonstrates the preliminary feasibility of auto-segmentation to support response evaluation in treated HCC.

## Introduction

Hepatocellular carcinoma (HCC) is the sixth most common type of cancer and the third leading cause of cancer-related mortality, with a notably high prevalence in East Asia (1). In the treatment of locally advanced HCC, there are several options, including local-ablative therapy [e.g., transcatheter arterial chemoembolization (TACE), external beam radiotherapy (EBRT), hepatic arterial infusion chemotherapy (HAIC)] as well as systemic therapies. According to the National Comprehensive Cancer Network (NCCN) guidelines for HCC, EBRT is recommended for patients with unresectable disease who are not transplant candidates, as well as for those with inoperable local disease, although the level of evidence remains Category 2B (2). Recent studies have reported definitive concurrent chemoradiotherapy (CCRT) with HAIC yields more favorable results compared with the non-CCRT group (3). Nevertheless, intrahepatic progression is common even after treatment, leading to liver failure and mortality. Thus, detection of locoregional progression and timely transition to alternative treatment options are critical for optimizing patient prognosis.

Computed tomography (CT) scanning is an essential modality for response evaluation, especially in HCC, which exhibits unique enhancement patterns on contrast-dynamic CT. Determining whether a tumor has progressed, remained stable, or responded to therapy is crucial for clinical decision making; however, such assessments are often subject to inter-observer variability between physicians and radiologists (4). The Response Evaluation Criteria in Solid Tumors (RECIST) are among the most widely adopted guidelines for treatment response assessment (5). In RECIST criteria, evaluation requires measurement of the “target lesion” based on its longest axis in axial slices. However, two major challenges exist; first, measuring tumor diameters requires significant professional expertise and is a time-consuming task, which is why the strict application of RECIST is often limited to clinical trials rather than routine clinical practice. Second, RECIST measurements are often subjective and may vary between radiologists, resulting in inconsistency.

Recently, with the advancement of deep learning tools, several studies have aimed to support treatment response evaluation with less effort. For instance, predict survival and cancer-specific outcomes, especially when combined with serial follow-up images (6). Similarly, Omar Ibrahim et al. introduced deep learning models for liver and tumor segmentation from CT scans; however, the validation accuracy for tumor segmentation remains suboptimal (7).

Developing reliable auto-segmentation models for treated HCC is particularly challenging for several reasons. HCC patients often suffer from severe liver cirrhosis, making the liver parenchyma coarse in appearance, and HCC lesions themselves are radiologically heterogeneous. Treated HCC lesions can take various forms depending on the treatment modality, such as scar tissue, necrotic areas, or fluid collections. Furthermore, residual embolic materials or artifacts from treatments like TACE or RFA can complicate image interpretation (8). Therefore, this study focuses on evaluating the size changes of the main HCC lesions using RECIST criteria after definitive CCRT in locally advanced, treatment-naïve HCC.

## Methods

### Study design and patient selection

Patients diagnosed with locally advanced HCC without nodal or distant metastasis who received definitive CCRT or RT between January 2016 and February 2021 at a single institution were identified. The medical records of all patients were retrospectively reviewed. All patients received intra-arterial 5-fluorouracil (FU) chemotherapy concurrently with 25 fractions of radiotherapy. The most commonly used dose fractionation was 2.4/1.8 Gy per fraction, employing a simultaneous integrated boost (SIB) technique to deliver a higher dose to the tumor. Tumor characteristics such as tumor size, extent, tumor markers, and survival status were collected from medical records and diagnostic imaging. Diagnostic CT image data were collected both at pre-treatment (CT_pre) and post-treatment (CT_post)—the first follow-up within 3 months. Most of the patients underwent contrast-enhanced CT using various Philips and Siemens CT scanners (Philips Healthcare, Siemens Healthineers). A specialized triple-phase liver protocol (arterial, portal venous, and delayed phases) was employed. For deep learning analysis, we utilized the portal venous phase CT images, which were reconstructed with an average slice thickness of 3.0 mm, and average voxel dimensions were approximately 0.7 × 0.7 × 3.0 mm^3^.

Tumor contour at both time points (Tumor_pre, Tumor_post) were manually delineated on portal phase CT images by a radiation oncologist and subsequently reviewed by a radiology expert. MRI scans were cross-referenced to further define tumor boundaries whenever available. The final ground truth contours were established through consensus between the two specialists. During the review process, major changes (e.g., significant modification of tumor boundaries) were required in 1% of the cases, while minor refinements (e.g., subtle adjustments of the contour edges) occurred in 8% of the cases. To develop a robust deep learning model while ensuring a reliable response evaluation, we categorized the patients into two distinct groups. First, the entire cohort (*n* = 83) was used for general model training and validation. Second, we defined a RECIST cohort (*n* = 63) by excluding 20 cases that did not meet the RECIST 1.1 criteria, such as those with extrahepatic lesions or extensive tumor thrombus extending to the inferior vena cava (IVC). This separation allowed for an optimal environment to train the segmentation model while specifically assessing its clinical utility for RECIST-based diameter measurements.

### Training and validation dataset

**Figure 1:**
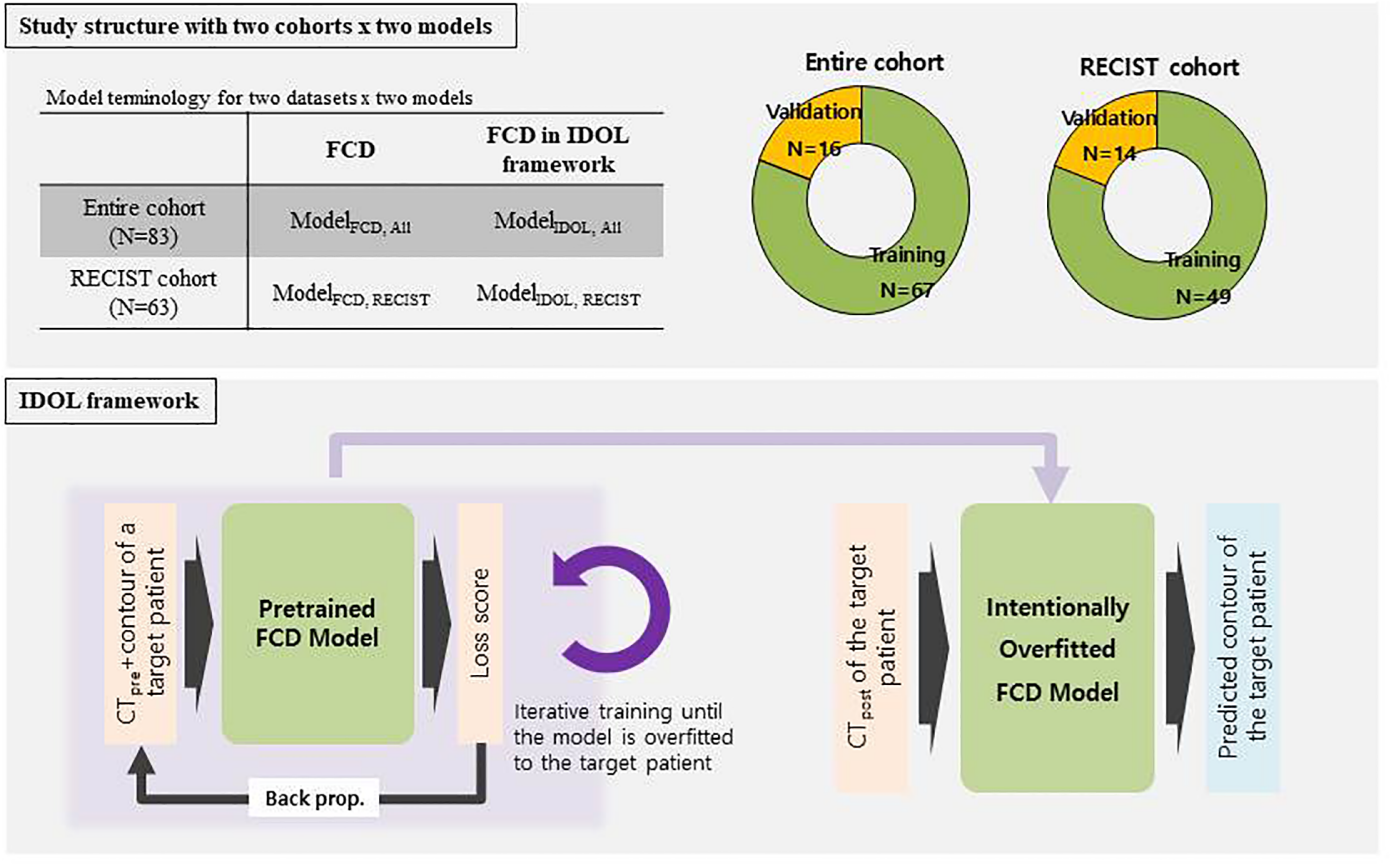
General workflow of FCD, IDOL model. At first, a generalized FCD model is produced by utilizing a training dataset of the patients. Next, optional task-driven augmentation by specific prior information produce a personalized model called IDOL. Tumor prediction was done 4 times for each datasets and deep learning models.

After image review and processing, a dataset of 83 patients was finalized. The entire cohort was divided into a training set (*n* = 67) and a validation set (*n* = 16). After excluding 20 RECIST-unfavorable cases, 63 patients remained and were divided into a training set (*n* = 49) and a validation set(*n* = 14). For each patient, dataset consisted of two time points as follows: {CT_pre, Tumor_pre} and {CT_post, Tumor_post}, where each element represented a radiotherapy planning CT, tumor contour on CT_pre, first follow-up CT, and tumor contour on CT_post, respectively. The dataset structure is illustrated in the upper panel of **Figure 1**.

### Fully convolutional DenseNet

For both the comparison model and the pretrained network used in the IDOL framework, a Fully Convolutional DenseNet (FCD)—a deep learning network for semantic segmentation—was employed. The input dimension of the FCD model was adjusted from two to three so that the entire 3D CT image can be processed. In addition, the original RGB-based three-channel input structure was converted to a single-channel gray-level medical image for this study (8). The network consisted of five dense blocks with 2, 3, 4, 5, and 7 layers and a growth rate of 12. Training was performed on a single NVIDIA GeForce GTX 1080 Ti GPU using the Adam optimizer with a polynomially decaying learning rate starting at 1 × 10^-^³ and a batch size of 1. The loss function combined cross-entropy, dual cross-entropy, and Dice coefficient loss, and data augmentation included random brightness scaling and uniform cropping.

### Intentional deep overfit learning framework

The intentional deep overfit learning (IDOL) framework, originally proposed for adaptive radiotherapy (ART), is task-agnostic and can therefore be applied to any task that meets its two-stage training strategy: (a) training a generalized model, that is, a conventional deep learning training, and (b) intentionally overfitting the generalized model to prior information of interest. In this study, we implemented the IDOL framework to generate a personalized model that automatically contours the tumor volume on the CT_post image (9).

For a pretrained network, we used the Fully Convolutional DenseNet as described above. Firstly, the pretrained FCD was retrained until overfitted to the target patient using prior information of CT_pre and the corresponding tumor contour. The prior information {CT_pre, Tumor_pre} was augmented through perturbation and repetition to achieve intentional overfitting. All hyperparameters were kept identical to the base model training. Adaptation was run for 350 epochs, after which the final checkpoint was used to predict the tumor contour on CT_post. Once an overfitted personalized model was generated, the model predicted the tumor contour using the CT_post image of the target patient. The Dice similarity coefficient (DSC) was calculated between the predicted tumor segmentation mask and the ground-truth segmentation mask derived from manual contouring. A schematic diagram of the model structure is shown in the lower panel of **Figure 1**.

### RECIST evaluation

After completion of the deep learning model analyses, maximal axial diameter was measured on both pre- and post-treatment CT images in the RECIST cohort according to RECIST guidelines. To evaluate the predictive performance, we calculated the 95% confidence intervals (CIs) for mean DSC values. A Wilcoxon signed-rank test was performed to compare the performance between the FCD and IDOL models. Statistical significance for RECIST-based response concordance was determined using McNemar’s test. Correlation between manual and predicted values was assessed using Spearman’s rank correlation analysis.

## Results

### Patient and tumor characteristics

Among the 83 patients diagnosed with HCC who received CCRT at a single institution, 67 were male and 16 were female. The age ranged from 33 to 82 years, with a median of 60 years. Forty-seven patients presented with α-fetoprotein (AFP) elevation, whereas 76 patients had protein induced by vitamin K absence (PIVKA)–II elevation. The maximal axial diameter decreased from an average of 118.6 mm before treatment to 96.9 mm after treatment, and the mean tumor volume decreased from an average of 672 cc to 427 cc. The 1-year locoregional free survival rate was 55.5%, and the 1-year overall survival rate was 64.4%. (**Table 1**).

**Table 1:** Patient and tumor characteristics.

Characteristics	No.	%
Sex		
Male	67	80.7
Female	16	19.3
Age (year)	Median 60 (33~82)	
AFP elevation (>10ng/ml)		
Yes	47	56.6
No	36	43.4
PIVKA-II elevation (>40mAU/ml)		
Yes	76	91.6
No	7	8.4
Diffuse PVTT		
Yes	20	24.1
No	63	75.9
Maximum axial diameter (CTpre)	118.6 ± 45.1 (mm)	
Maximum axial diameter (CTpost)	96.9± 43.8 (mm)	
Tumor volume (CTpre)	672.0 ± 623.9 (cc)	
Tumor volume (CTpost)	426.9 ± 508.7 (cc)	
1yr-LRFS	55.50%	
1yr-Overal survival	64.40%	

AFP, α-fetoprotein; PIVKA, Proteins Induced by Vitamin K Absence; LRFS, Loco-regional progression Free Survival.

### FCD model and IDOL model in the entire cohort

In the entire cohort, the FCD model was applied to predict tumor contours on both CT_pre and CT_post images, while the IDOL model was generated by incorporating each case’s CT_pre data to improve performance. Sixty-seven cases (134 CT images in total) were used as the training set, and 16 cases were used for validation. For CTpre images, DSC was calculated between the predicted tumor segmentation mask and the ground-truth segmentation mask derived from manual contouring for both models. DSCs between manual contour and auto-segmented results from the IDOL model for CT_post images were also calculated (**Supplementary Table 1A**). In the 16 validation cases, the mean DSC of CT_pre was 0.53 using the FCD model and 0.33 for CT_post. In contrast, the IDOL model improved CT_post DSC to 0.49. The IDOL model demonstrated superior performance in several challenging cases (e.g., Cases 1 and 7 in **Supplementary Table 1**) where the conventional FCD model failed to provide any valid tumor predictions. However, in certain complex scenarios such as Case 6, both the FCD and IDOL models failed to accurately predict the post-treatment tumor contours. In Case 6, the patient initially presented with an extensive tumor thrombus extending along the IVC. Following treatment, there was a rapid and significant reduction in tumor size.

### FCD model and IDOL model in the RECIST cohort

In the RECIST cohort, 49 cases were used as the training set and 14 cases as the validation set for developing the FCD and IDOL models within the RECIST cohort. The DSCs between manual and predicted contours are summarized in **Table 2**. The mean DSC for CTpre was 0.61 with the FCD model. For CT_post, the mean DSCs were 0.53 and 0.63 for the FCD and IDOL models, respectively. A cross-comparison of DSCs between cohorts and models is summarized in **Table 3**.

**Table 2:** Dice similarity coefficient (DSC) of RECIST cohort per FCD and IDOL model.

Model	FCD/CTpre	FCD/ CTpost	IDOL/ CTpost
Case	DSC	DSC	DSC
**1**	0.45	0.07	0.21
**2**	0.77	0.57	0.52
**3**	0.65	0.69	0.78
**4**	0.46	0.29	0.62
**5**	0.90	0.75	0.83
**6**	0.82	0.80	0.85
**7**	0.61	0.16	0.58
**8**	0.55	0.42	0.68
**9**	0.92	0.87	0.90
**10**	0.81	0.70	0.57
**11**	0.16	0.14	0.64
**12**	0.26	0.54	0.57
**13**	0.83	0.76	0.63
**14**	0.32	0.64	0.50
Mean	0.61	0.53	0.63

RECIST, Response Evaluation Criteria in Solid Tumours; FCD, Fully Convolutional DenseNet; IDOL, Intentional deep overfit learning.

**Table 3:** Comparison of mean dice similarity coefficient of 2 by 2 model.

Model	Patients group	Mean DSC	
		CTpre	CTpost
FCD	All	0.53	0.33
IDOL	All	–	0.49
FCD	RECIST	0.61	0.53
IDOL	RECIST	–	0.63

DSC, mean dice similarity coeffcient; FCD, Fully Convolutional DenseNet; IDOL, Intentional deep overfit learning.

### Predicted maximum axial diameter (RECIST) and tumor volume

The maximal axial diameters for 14 validation cases in the RECIST cohort were measured for both manual and IDOL model-predicted contours. Measurements and post-treatment changes are summarized in **Table 4**. The average maximal axial diameter of manually delineated tumors was 118.2 mm before treatment and decreased to 90.4 mm after treatment, corresponding to an average reduction of 19.9% (range, −52.6% to +2.1%). The average predicted value by the auto-segmentation model was 97.6 mm, differing by approximately 6.1% from the manual measurement. The model performance (DSC) was positively and significantly correlated with the pre-treatment tumor maximal axial diameter (*p* < 0.01, *ρ* = 0.842), suggesting higher model accuracy in cases with larger baseline tumor dimensions.

**Table 4A:** Maximal axial diameters and differences after treatment by manually drawn contour and predicted contour by Model (IDOL, RECIST).

	Maximal axial diameter			Difference after treatment			
Case ID	Manual Contour		Predicted Contour	Manual Contour(%)	RECIST Classification	Predicted Contour(%)	RECIST Classification
	CTpre(mm)	CTpost(mm)	CTpost(mm)				
**1**	113.5	64.6	80.8	-28.4	SD	-10.5	SD
**2**	151.8	74.3	80.4	-52.6	PR	-48.7	PR
**3**	129.3	87.5	84.8	1.9	SD	-1.2	SD
**4**	100.1	61.8	63.1	-33.7	PR	-32.3	PR
**5**	178.0	159.0	170.1	-2.9	SD	3.9	SD
**6**	152.5	138.5	147.7	-8.8	SD	-2.8	SD
**7**	100.0	91.7	85.5	-5.3	SD	-11.8	SD
**8**	95.6	81.1	82.0	-7.8	SD	-6.8	SD
**9**	156.8	144.0	145.0	-0.3	SD	0.4	SD
**10**	114.3	80.4	109.1	-36.0	PR	-13.2	SD
**11**	128.9	47.1	48.9	-14.3	SD	-11.0	SD
**12**	95.9	68.2	116.3	-43.8	PR	-31.9	PR
**13**	143.1	114.4	126.8	-25.9	SD	-17.9	SD
**14**	22.6	25.3	26.3	2.1	SD	6.3	SD
Mean	118.2±38.3	88.4±37.6	97.6±41.5				
p value			0.032*				

RECIST, Response Evaluation Criteria in Solid Tumours; IDOL, Intentional deep overfit learning; PR, Partial Response; SD, Stable Disease.

*p-value between maximal axial diameter between Manual and Model prediction, using paired T-test.

**Table 4B:** Statistical summary of RECIST Classification by both manual contour and IDOL model prediction.

Manual Classification	IDOL model prediction		Total (No.)
	PR (No.)	SD (No.)	
PR	3	1	4
SD	0	10	10
Total	3	11	14

Sensitivity : 0.75, Spescificity : 1.00, AUC = 0.875.

p=1.00 (McNemar’s Test).

According to RECIST criteria, 4 patients exhibited partial response (PR) and 10 had stable disease (SD). Although the model showed a statistically significant difference (*p* = 0.032) in maximal axial diameters, but it demonstrated reliable diagnostic performance for PR prediction, achieving a sensitivity of 75% and a specificity of 100% and an AUC of 0.875. The lack of statistical significance in McNemar’s test (*p* = 1.000) further confirms that the model-derived RECIST classifications are highly consistent with those of human experts.

The IDOL model predicted three cases as PR and one true PR case as SD, which showed a −36.0% reduction in maximum axial diameter manually but −13.2% according to the model prediction. The single discordance case was characterized by multiple necrotic lesions merging in the inferior portion of liver segment 6. The IDOL model’s prediction for the post-treatment image extended into the adjacent segment 5 parenchyma, resulting in a diameter reduction of only 13.2% (SD), while the manual contour yielded a reduction of 36% (PR). (**Tables 4a, b**).

Regarding the IDOL model’s performance after treatment, there was a trend toward overestimation compared to the manually drawn contours. Specifically, the model-predicted maximal axial diameter showed an average increase of approximately 9.2 mm over the manual measurements. In terms of tumor volume, the model’s predictions were, on average, 119.8 cc larger than the ground truth.

## Discussion

The purpose of this study was to assist post-treatment response evaluation using deep learning-based auto-segmentation. In general, HCC is considered a challenging target for auto-segmentation by deep learning models due to the irregularity of the background liver and the heterogeneity of the tumor itself (10). Furthermore, in patients with locally advanced disease, the difficulty is further compounded by the accompanying diffuse PVTT and tumor irregularity among hepatic vasculature. In this study, through IDOL analysis, we attempted to improve the performance of automated tumor localization and size measurement to a clinically useful level of accuracy.

In the analysis of the entire 83-patient cohort, the general FCD model demonstrated relatively low performance (**Supplementary Table 1**). Previous studies on liver tumor auto-segmentation, including mostly metastatic liver lesions and few HCC tumors, have reported DSCs of approximately 0.6–0.7 (10). More recently, new models such as nnU-NET or transformer-based models (e.g., SwinUNETR, ResU-nET) were developed for tumor segmentation of HCC (11, 12). Although these models are based on single, small lesions suitable for TACE therapy, the DSC performance was higher than 0.7.

In contrast, our cohort featured predominantly large, irregularly shaped tumors, often accompanied by extensive PVTT showing poorly defined tumor boundaries on dynamic CT scans. The predictive performance of the FCD model was further reduced for CT_post images, particularly in cases with changes in PVTT lesions that were difficult to delineate. In contrast, the IDOL model, which was overfitted to each individual patient’s CT and tumor contour, showed improved accuracy with an increasing average DSC of 0.49.

Higher performance was observed within the RECIST cohort. The FCD model achieved a mean DSC of 0.61, similar to values reported in prior studies. After treatment, the mean DSC decreased to 0.53; however, the IDOL model improved performance to a mean DSC of 0.63—the highest among models and comparable to pre-treatment accuracy.

**Figure 2:**
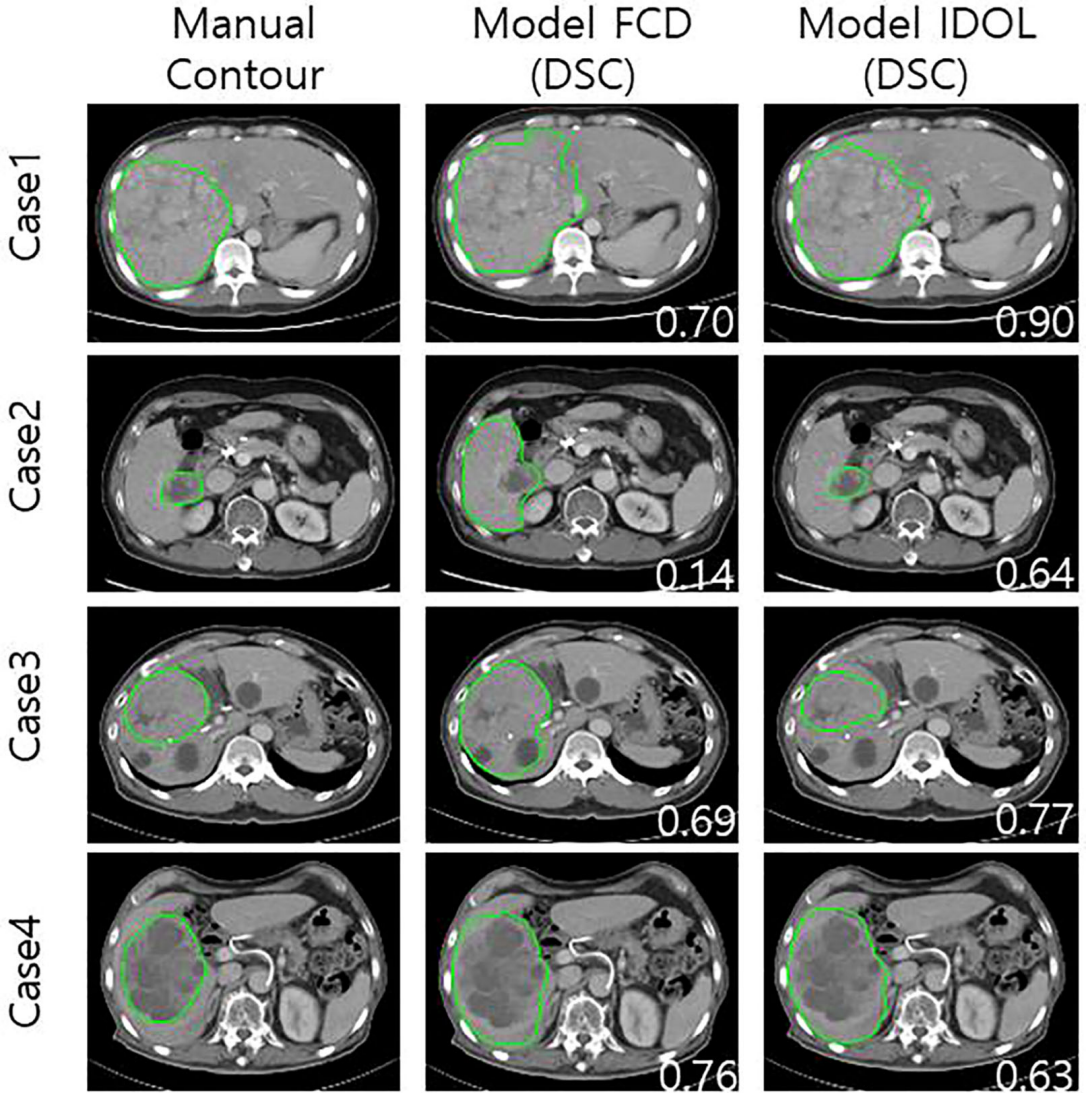
Examples of tumor prediction model and comparison with manually drawn contour in RECIST cohort. Tumor contours on CT scans after treatment are arranged in rows according to manual contour and model FCD & IDOL.

The FCD model tended to overestimate tumor boundaries, particularly at the superior and inferior poles, whereas the IDOL model produced tighter, more anatomically accurate contours (**Figure 2**: Cases 2 and 3). Distant liver metastases were not detected in model predictions.

Within the IDOL framework, both pre-treatment imaging and the corresponding tumor segmentations were utilized for model training. When applied to post-treatment images, the model facilitated tumor segmentation but tended to over-segment peritumoral areas such as regions around tumors, such as post-treatment edema. This tendency resulted in general overestimation of tumor volumes and lower DSC scores on post-treatment CT scans. (**Figure 2**: Case 4).

To confirm whether RECIST response evaluation by deep learning was reasonable, maximal axial diameter was evaluated using the IDOL (RECIST) model. The model achieved reasonable prediction accuracy, with an absolute difference of approximately 9% in maximal axial diameter compared with manual measurements. We observed that the percentage error in total tumor volume (22.4%) was notably higher than the average increase in maximal axial diameter (approximately 10.4%). This discrepancy suggests that while the model accurately predicts the viable core of the tumor, it tends to overestimate the boundaries by including the adjacent treated liver parenchyma. This overestimation of the peripheral boundaries after treatment is clearly demonstrated in the representative images of Case 4 in **Figure 2**, where the model’s predicted contour extends slightly beyond the actual tumor margin into the peritumoral area. This phenomenon is likely attributable to inflammatory reactions in areas that received low-dose radiation during CCRT, which can alter the radiomic features of the peritumoral liver parenchyma. We anticipate that these discrepancies could be mitigated in future studies by incorporating multi-phase CT or MRI-based model building rather than relying solely on a single portal phase. Furthermore, employing modified RECIST (mRECIST) to focus specifically on enhancing viable components would likely refine the model’s ability to distinguish between active tumor and treatment-related inflammatory changes.

Regarding clinical implementation of the IDOL model, a two-step workflow is required, as it necessitates pre-treatment tumor contours. For HCC patients treated with CCRT, this model can be integrated into the existing clinical pipeline without additional effort, as accurate tumor segmentation is already a standard part of the radiation treatment planning process. For broader applications in general oncology—where manual contours may not be readily available—the practical solution lies in combining the IDOL model with an automated pre-treatment segmentation step. Although we evaluated the FCD model for this purpose, the workflow remains flexible; the first step can be substituted with any state-of-the-art segmentation model to optimize the overall accuracy of RECIST prediction in clinical practice.

This study has several limitations. First, we did not perform external validation due to constraints in patient data availability. Second, a discrepancy exists between the ground truth tumor contour and the dataset used for model training. Due to the nature of HCC, delineating boundaries of locally advanced cases, especially with PVTT or microinvasion, is challenging on a single phase using dynamic CT alone. To overcome this uncertainty, all available CT and MRI phases were used to create a manual tumor contour, which was reviewed by an expert liver radiologist to maximize ground truth quality. On the other hand, since the deep learning model was trained only on the single (portal) phase of CT, there was a limitation to predicting true tumor boundaries. Because of the characteristics of HCC, post-treatment liver shrinkage and changes in adjacent bowel or gallbladder regions negatively affected model performance on post-treatment images. Finally, the IDOL framework’s dependency on pre-treatment labels may limit the applicability of the model to populations not undergoing radiotherapy.

In conclusion, the personalized IDOL model trained using pre-treatment data from HCC patients was able to predict post-treatment tumors more accurately than base architecture models. The predicted contours tended to include peripheral liver tissue near the tumor poles but demonstrated better accuracy in the main tumor slices, which are most relevant for RECIST-based evaluation. Although this study focused on patients treated with CCRT, who often present with more irregular tumors and greater challenges for segmentation, the predictive performance (DSC) of the baseline FCD model was relatively inferior compared with more recent state-of-the-art models. Nevertheless, this study specifically attempted to predict post-treatment tumor morphology, and its findings provide preliminary evidence that such an approach can be extended and applied to other deep learning architectures. In this regard, our results offer meaningful insight into the feasibility of post-treatment tumor segmentation and may serve as a foundation for future model development.

We anticipate that such tools may aid clinical decision-making and reduce the workload for clinicians. Future studies incorporating multi-phase CT and MRI and combinations with state-of-the-art models are warranted to overcome current limitations and improve prediction accuracy, particularly for cases with diffuse PVTT or high tumor heterogeneity.

## Data Availability

The original contributions presented in the study are included in the article/**Supplementary Material**. Further inquiries can be directed to the corresponding author.
