# Supplementary material for: Deep learning–based auto-segmentation and RECIST evaluation after concurrent chemoradiotherapy in locally advanced hepatocellular carcinoma patients
Source: Front Oncol. 2026 Mar 30;16:1775269. doi: 10.3389/fonc.2026.1775269 (PMC13070800; doi:10.3389/fonc.2026.1775269)
Supplement: Supplementary file 1 [file Table1.docx]

Supplementary Table 1. Dice similarity coefficient (DSC) of entire cohort per FCD and IDOL model

| Model | FCD,All | FCD,All | IDOL,All |
| --- | --- | --- | --- |
| CT | CTpre | CTpost | CTpost |
| **1** | 0.56 | 0.02 | 0.37 |
| **2** | 0.84 | 0.59 | 0.66 |
| **3** | 0.36 | 0.02 | 0.27 |
| **4** | 0.29 | 0.21 | 0.39 |
| **5** | 0.17 | 0.02 | 0.36 |
| **6** | 0.74 | 0.00 | 0.01 |
| **7** | 0.87 | 0.17 | 0.58 |
| **8** | 0.42 | 0.54 | 0.49 |
| **9** | 0.71 | 0.55 | 0.80 |
| **10** | 0.20 | 0.25 | 0.34 |
| **11** | 0.81 | 0.50 | 0.46 |
| **12** | 0.40 | 0.33 | 0.33 |
| **13** | 0.42 | 0.30 | 0.36 |
| **14** | 0.86 | 0.81 | 0.94 |
| **15** | 0.76 | 0.65 | 0.81 |
| **16** | 0.13 | 0.38 | 0.65 |
| Mean | 0.53 | 0.33 | 0.49 |

***Abbreviations:*** DSC : mean dice similarity coeffcient, FCD : Fully Convolutional DenseNet. IDOL : Intentional deep overfit learning

Supplementary Table 2. Tumor volume by manually drawn contour and predicted contour by Model (RECIST, FCD & IDOL)

|  | Tumor volume(cc) | | |
| --- | --- | --- | --- |
| Case | Manual(CTpost) | Model FCD | Model IDOL |
| **1** | 27.9 | 815.5 | 233.8 |
| **2** | 517.4 | 1123.2 | 338.7 |
| **3** | 388.4 | 620.3 | 251.9 |
| **4** | 150.6 | 868.8 | 125.2 |
| **5** | 1261.5 | 2024.3 | 1749.3 |
| **6** | 1331.7 | 1595.9 | 1734.4 |
| **7** | 284.7 | 26.5 | 122.9 |
| **8** | 166.3 | 253.5 | 128.4 |
| **9** | 1796.1 | 2033.5 | 2032.5 |
| **10** | 232.3 | 304.2 | 529.4 |
| **11** | 74.3 | 846.9 | 40.8 |
| **12** | 651.9 | 436.1 | 594.6 |
| **13** | 609.7 | 982.8 | 1283.4 |
| **14** | 6.3 | 11.6 | 16.0 |
| Mean | 535.6 | 853.1 | 655.8 |

***Abbreviations:*** FCD : Fully Convolutional DenseNet. IDOL : Intentional deep overfit learning
